# Oral antibiotics relieve allergic asthma in post-weaning mice *via* reducing iNKT cells and function of ADRB2

**DOI:** 10.3389/fimmu.2022.1024235

**Published:** 2022-10-25

**Authors:** Na Li, Jie Chen, Sitao Xie, Meng Zhang, Tianyun Shi, Yanchao He, Zhijun Jie, Xiao Su

**Affiliations:** ^1^ Department of Pulmonary and Critical Care Medicine, Shanghai Fifth People’s Hospital, Fudan University, Shanghai, China; ^2^ Department of Medicine, Respiratory, Emergency and Intensive Care Medicine, The Affiliated Dushu Lake Hospital of Soochow University, Suzhou, China; ^3^ Unit of Respiratory Infection and Immunity, Institute Pasteur of Shanghai, Chinese Academy of Sciences, Shanghai, China

**Keywords:** asthma, asthma tolerance, antibiotics, iNKT cells, ADRB2^+^ dendritic cells

## Abstract

The role of normal gut microbiota in asthma or ovalbumin (OVA)-induced asthma tolerance (OT) remains unclear. Here, we established mouse models of asthma and OT followed by 2 weeks of antibiotic treatment, to clear the gut microbiota. Antibiotic treatment was found to alleviate allergic asthma accompanied with a reduction of invariant natural killer (iNKT) cells. By RNA-seq analysis, we found that β-adrenergic receptor (ADRB) genes, including *Adrb1*, *Adrb2*, and *Adrb3*, were downregulated in asthmatic lungs, but these changes were reversed in OT lungs. Moreover, *Adrb2* and *Adrb3* were significantly upregulated in asthmatic lungs after antibiotic treatment. Surprisingly, blocking ADRB with propranolol relieved allergic asthma while reducing T helper 2 (Th2) and Treg cell numbers. Further analyses using flow cytometry and immunofluorescence showed that the protein expression level of ADRB2 was higher in asthmatic lungs than that in the control and OT lungs. Notably, dendritic cells (DCs), especially the ADRB2^+^ DCs, were increased in asthmatic lungs compared to that in the control and OT lungs. In addition, ADRB2^+^ DCs were significantly reduced following the administration of the ADRB2-specific antagonist ICI118551. Our findings suggest that antibiotic treatment can alleviate OVA-induced allergic asthma *via* reducing the frequency of iNKT cells and function of ADRB2.

## Introduction

Asthma is one of the most common chronic inflammatory diseases worldwide, affecting approximately 300 million people (1). Allergic asthma is a type of allergic disease and immune tolerance prevents antigen-induced pathological reactions. The establishment of immune tolerance is important for asthma. Oral immunotherapy is one of the important means of treating allergic diseases (2). Several studies have found that the establishment of oral immune tolerance originates from the intestinal immune system (3, 4), and relies on CCR7^+^CD103^+^ DCs to transport antigens to mesenteric lymph nodes (MLNs) (5). However, the mechanisms underlying asthma and oral immune tolerance are complex. Using 4-week-old BALB/c mice provided with ovalbumin (OVA) - containing drinking water, we recently established an OVA - induced asthma tolerance (OT) mouse model (6, 7). Here, we explored the effects of antibiotics on the development of asthma and established OT by providing mice with antibiotic drinking water, which is capable of altering their gut microenvironment.

At present, there is still no consensus on whether antibiotics promote or inhibit the development of asthma. Maternal antibiotic treatment during pregnancy that decreases cecal short-chain fatty acid, has been found to increase asthma severity in the offspring in a dose-dependent manner (8). When mice are provided with drinking water containing ampicillin, gentamicin, metronidazole, neomycin sulfate, and vancomycin for four weeks, the plasma IgE levels and basophils are increased, leading to increased inflammation due to house dust mite (HDM) (9). Another study has also found similar results that vancomycin treatment before weaning can also worsen asthma symptoms, accompanied by an increase in plasma IgE levels and a decrease in regulatory T cells (10). Conversely, many studies found that antibiotic treatment might relieve allergic asthma. Treatment with ceftriaxone or vancomycin significantly reduces serum total IgE levels in non-sensitized mice at postnatal day 21. After four rounds of intraperitoneal sensitization, the plasma OVA-specific- and total IgE levels in the vancomycin-treated mice are decreased compared to those in the controls (11). Studies in post-weaning mice have also found that the commensal gut microbiota through the NLRP3/IL-1β pathway promote the development of OVA-induced asthma, which can be alleviated by antibiotic use (12). Likewise, antibiotic treatment of mice with lipopolysaccharide/elastase-induced lung inflammatory disease improves lung function and reduces inflammation, which implies that host-microbial cross-talk promotes inflammation and hence might cause the chronicity of inflammatory lung diseases (13).

Invariant natural killer T (iNKT) cells are innate immune cells; they are affected by antibiotics. Several studies have shown that lipids produced by gut microbes can effectively activate iNKT cells (14, 15). The number of iNKT cells are significantly reduced after the gut microbiota are cleared (16, 17). When gut microbiota composition is altered or absent, iNKT cells do not expand properly and do not reach a normal functional state, on antigen stimulation (18). Classification and function of iNKT cells are similar to that of T helper 2 (Th2) cells (19). α-Galactosylceramide (α-Galcer) administration activated iNKT cells increases OVA-induced Th2 inflammatory responses in the airways of mice (20). Therefore, antibiotic treatment is likely to affect the development of asthma by disrupting the development and function of iNKT cells.

As receptors activated by epinephrine (Epi) or norepinephrine (NE), beta adrenergic receptors (ADRB) include ADRB1, ADRB2 and ADRB3 (21). ADRB2 has been found to relax bronchial smooth muscle (22), short or long agonists of ADRB2 are also commonly used clinically in the treatment of asthma as a bronchodilator (23). However, the long-term use of ADRB2 agonists in patients with asthma may sometimes worsen their condition. Clinically, it is necessary to simultaneously use anti-inflammatory drugs such as glucocorticoids, to improve the symptoms (24). Two pathways have been found to activate ADRB2, of which the Gs-cAMP-mediated canonical pathway is believed to activate ADRB2 in smooth muscle cells to promote bronchodilation. The other pathway is considered as a pro-inflammatory role in asthma models (25). The use of ADRB2 agonists also exacerbates inflammatory responses (26). Additionally, chronic activation of ADRB2 and ADRB3 promotes neuroinflammation by increasing immune cell activation and proinflammatory cytokine production (27). ADRB2 blockers, such as propranolol and metoprolol, reduce pulmonary inflammatory response in patients with COVID-19 when acute respiratory distress syndrome occurs, suggesting that ADRB2 might be involved in the inflammatory response (28, 29). The use of nadolol can prevent the progression of OVA-induced asthma phenotype (30). Some ADRB2 antagonistic blockers may relieve asthma by blocking ADRB2 and maintaining the stability of inactive receptors (31).

As antigen-presenting cells, DCs can present antigen to T cells to initiate protective pro-inflammatory responses and are a key starting point for adaptive immunity (32, 33). Studies have found that DCs express functional ADRB2 which is associated with inflammatory factors (34–36).

Here, we hypothesize that oral antibiotics affect allergic asthma in weaned mice by altering the function of iNKT cells and ADRB2. First, we investigated whether the use of antibiotics relieved asthma or impaired oral immune tolerance, which was later established. Changes in iNKT and ADRB2^+^ cells were then detected. In particular, we unexpectedly found that blocking ADRB2 can alleviate asthma, and the antagonistic effect of ADRB2 can reduce ADRB2^+^ DCs, suggesting that ADRB2 is a causative factor in asthma. Therefore, antibiotic therapy can relieve asthma by reducing the function of iNKT cells and ADRB2.

## Materials and methods

### Animal

4-week-old female specific-pathogen-free Balb/c mice were purchased from Beijing Weitong Lihua Laboratory Animal Technology Co., Ltd. (Beijing, China) and housed in the Pasteur Institute of Shanghai, Chinese Academy of Sciences (Shanghai, China). During the experiment, the mice were in an environment with alternating dark/light every 12 h and had free access to sufficient food and autoclaved drinking water. The animal experiments were reviewed and approved by the Laboratory Animal Welfare Ethics Committee of the Pasteur Institute of Shanghai, Chinese Academy of Sciences.

### Establishing asthma and OT mouse models

**Figure 1:**
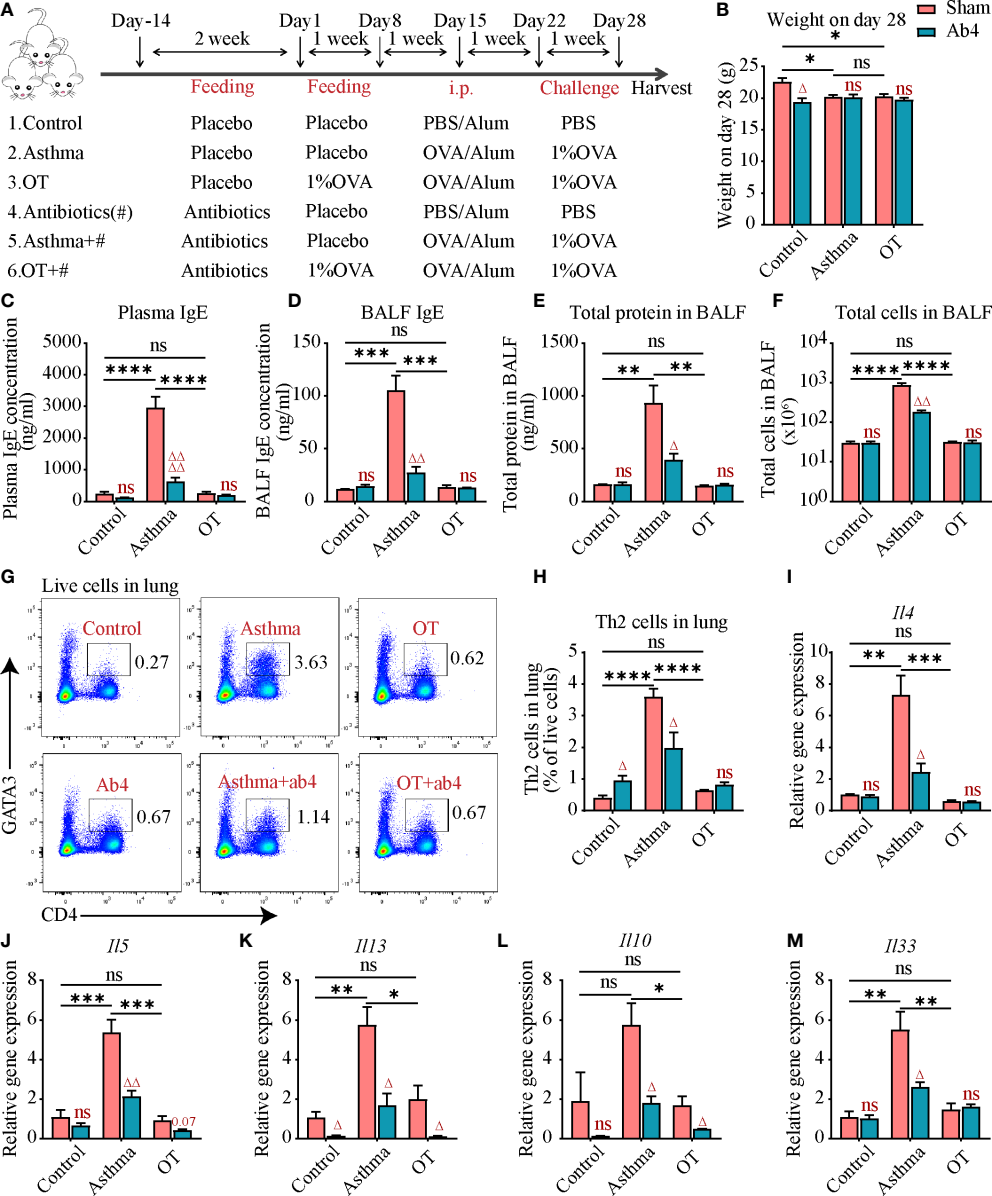
Antibiotic treatment alleviates allergic asthma. **(A)** Experimental protocol for establishing asthma and OT mouse models with or without oral antibiotics. # stands for antibiotics. The detailed procedures have been described in the Materials and Methods. **(B)** Body weight of mice in each group on day 28. **(C)** Plasma IgE levels assessed by ELISA (data were pooled from 3 independent experiments, N=3-4 in each independent experiments). **(D)** Bronchoalveolar lavage fluid (BALF) IgE levels assessed by ELISA. **(E, F)** Total number of cells and total protein levels in BALF. **(G, H)** Flow cytometry analysis for CD4^+^GATA3^+^ Th2 cells in lung tissue. **(I–M)** Gene expression levels of *Il4, Il5, Il13, Il10* and *Il33* respectively in the whole lung assessed by real-time qPCR. Results are from one representative experiment of at least two independent experiments (N = 3-4 in A-B and D-M). All bars in this figure indicate mean ± SEM. * P < 0.05, ** P < 0.01, *** P < 0.001, and **** P < 0.0001 represent multiple comparisons of control, asthma and OT groups (corrected by one-way ANOVA with Tukey’s test). ^△^ P < 0.05, ^△△^ P < 0.01, and ^△△△△^ P < 0.0001 represent comparisons of adjacent bars of Ab4 and sham in each group (unpaired two-tailed Student’s t-test); ns, not significant.

As previously described (6), female Balb/c mice were divided into three groups (3-4 per group): (i) control group: OT was not induced in the first week, intraperitoneal injection (50 µl aluminum adjuvant in 75 µl phosphate-buffered saline [PBS]) was performed on day 15, and aerosol challenge (PBS) was performed on days 22–28; (ii) asthma group: OT was not induced in the first week, intraperitoneal sensitization (50 µg OVA and 50 µl aluminum adjuvant in 70 µl PBS) was performed on day 15, and aerosol challenge (10 mg/ml OVA in PBS) was performed on days 22–28; and (iii) OT group: OT was induced (using 10 mg/ml OVA dissolved in autoclaved drinking water) on days 1–7, intraperitoneal sensitization (50 µg OVA and 50 µl aluminum adjuvant in 70 µl PBS) was performed on day 15, and aerosol challenge (10 mg/ml OVA in PBS) was performed on days 22–28 (**Figure 1A**).

### Antibiotic administration

Neomycin sulfate, ampicillin sodium and metronidazole were dissolved in sterile water at a concentration of 1 mg/ml, vancomycin hydrochloride was dissolved in sterile water at a concentration of 0.5 mg/ml (37–39). All antibiotics were purchased from Sangon Biotech (Shanghai, China) and antibiotic drinking water which was changed every three days was freely available to 4-week-old mice for 2 weeks (**Figure 1A**).

### Collection of BALF

BALF was collected via a tracheal incision in a process that involved washing the lungs three times with 1 ml PBS. After centrifugation at 500 g for 15 min at 4°C, BALF cells were collected from the sediment for counting and the supernatant was stored in a -80°C freezer prior to detecting the total protein levels (Pierce™ bicinchoninic acid [BCA] protein assay kit, Thermo Scientific, MA, USA) and IgE levels (as described below).

### Collection of plasma

On day 28 of models, the mice were anesthetized and about 0.5–1 ml of cardiac blood was obtained, using ethylenediaminetetraacetic acid (EDTA) to prevent coagulation. After centrifugation at 1500 g for 10 min at 4°C, the upper layer (plasma) was transferred into a 1.5-ml tube and stored in a -80°C freezer.

### IgE levels

The IgE levels in the BALF and plasma were detected using a mouse IgE enzyme-linked immunosorbent assay (ELISA) set (BD OptEIA™, BD Biosciences, NJ, USA) following the supplier’s instructions. A standard curve was drawn using pre-prepared standards.

### Real-time quantitative PCR (qPCR)

Total RNA was extracted using TRIzol reagent (Invitrogen, CA, USA). After the RNA concentration was measured, cDNA was synthesized from 1 µg RNA using a reverse transcriptase kit (Hifair III 1st Strand cDNA Synthesis SuperMix for qPCR (gDNA digester plus), Yeasen, Shanghai, China) according to the manufacturer’s protocol. Next, real-time qPCR was performed using an Applied Biosystems™ QuantStudio™ 6 Flex (Thermo Scientific, USA). A two-step method was used and the thermocycling parameters were as follows: 95°C for 1 min and then 40 cycles of 95°C for 10 s and 60°C for 10 s, with the default melting curve program. The relative expression levels of corresponding genes were determined by the 2^-△△^ CT method and normalized by GAPDH. The sequences of the forward and reverse primers were as follows:

*Il4*: 5’ -AAGAACACCACAGAGAGTGAGCTC-3’(forward)

and 5’-TTTCAGTGATGTGGACTTGGACTC-3’(reverse)

*Il5*: 5’ -GAGCACAGTGGTGAAAGAGACCTT-3’(forward)

and 5’-ATGACAGGTTTTGGAATAGCATTT-3’(reverse)

*Il13*: 5’ -AAAGCAACTGTTTCGCCACG-3’(forward)

and 5’-CCTCTCCCCAGCAAAGTCTG-3’(reverse)

*Gapdh*: 5’ -AGGTCGGTGTGAACGGATTTG-3’ (forward)

and 5’-TGTAGACCATGTAGTTGAGGTCA-3’(reverse)

*Il33*: 5’ -TCCTTGCTTGGCAGTATCCA-3’(forward)

and 5’-TGCTCAATGTGTCAACAGACG-3’(reverse)

*Il10*: 5’ -CCCTTTGCTATGGTGTCCTT-3’(forward)

and 5’-TGGTTTCTCTTCCCAAGACC-3’(reverse)

*Adrb1*: 5’ -CTCATCGTGGTGGGTAACGTG-3’(forward)

and 5’-ACACACAGCACATCTACCGAA-3’(reverse)

*Adrb2*: 5’ -GGGAACGACAGCGACTTCTT-3’(forward)

and 5’-GCCAGGACGATAACCGACAT-3’(reverse)

*Adrb3*; 5’ -GGCCCTCTCTAGTTCCCAG-3’(forward)

and 5’-TAGCCATCAAACCTGTTGAGC-3’(reverse)

### Single-cell suspensions

The entire lungs were digested with 50 µg/ml Liberase (Roche, Basel, Switzerland) and 1 µg/ml DNase I at 37°C for 45 min with shaking (40). The digested lung tissue was centrifuged at 350 × g for 7 min at 4°C. Cells were collected after lysing the erythrocytes for 7 min using Red Blood Cell Lysis Buffer (Beyotime, Shanghai, China). Finally, a single-cell suspension was obtained using a 70-µM cell strainer (BD Biosciences, USA). Similarly, hilar lymph nodes (HLNs) and mesenteric lymph nodes (MLNs) were digested with 1 mg/ml Collagenase IA at 37°C for 30 min. A 5-ml syringe was used to gently grind the digested HLNs and MLNs in a 40-µM cell strainer. Cells were collected for flow cytometry.

### Flow cytometry

Purified rat anti-mouse CD16/CD32 (Clone 2.4G2, BD Biosciences, USA) was used to block Fc fragments on immune cells in the single-cell suspensions for 15 min at 4°C. To identify viable cells before surface staining, the cells were stained with Fixable Viability Stain 780 (BD Biosciences, USA) in sodium azide-free and protein-free Dulbecco’s phosphate-buffered saline (1:100) for 15 min at room temperature. Next, the single-cell suspensions were incubated with the following monoclonal antibodies against surface antigens for 30 min at 4°C in the dark: APC anti-mouse CD11c Antibody (N418), APC anti-mouse CD4 Antibody (GK1.5), FITC anti-mouse I-A/I-E Antibody (M5/114.15.2), APC-Cy™7 Rat Anti-Mouse CD45 (30-F11), PE Rat Anti-Mouse Siglec-F (E50-2440) (all from Biolegend, CA, USA). PerCP-Cy™5.5 Hamster Anti-Mouse TCR β Chain (H57-597), BV605 Hamster Anti-Mouse CD3e (145-2C11) (all from BD Biosciences, USA). Mouse Cd1d Tetramer(a-Galcer loaded)-PE (from Medical & Biological Laboratories Co., Ltd.).

Primary antibodies Rabbit polyclonal antibody to Beta-2 Adrenergic Receptor (PA3116, Ambar) was used for indirect surface staining for 2 hours at 4°C in the dark. Goat Anti-Rabbit IgG (H&L) - Alexa Fluor 647 was used as a fluorescent secondary antibody for 1 hour at 4°C in the dark. These two antibodies were purchased from Abmar.

A transcription factor buffer set (BD Biosciences, USA) was used for intranuclear staining. Nuclear membranes were fixed and permeabilized with 1× fix/perm buffer for 50 min at 4°C. The following fluorescent anti-nuclear proteins antibodies (against FoxP3 and GATA3) were added to cells suspended with 80–100 µl of 1× perm/wash buffer at 2–8°C for 40–50 min in the dark: PE anti-mouse FOXP3 Antibody(MF-14), BV421 Mouse Anti-GATA3(L50-823) (from Biolegend).

All samples were passed through a 40-µM cell strainer prior to flow cytometry. Data were acquired with an LSRFortessa™ flow cytometer (BD Biosciences, USA) and processed with FlowJo software (TreeStar, Ashland, OR, USA).

### Immunofluorescence of lung tissue

On day 28 of models, Balb/c mice were euthanized, and their entire lungs were removed and placed in 4% paraformaldehyde. Single-labeled immunofluorescence staining of ADRB2 in the lung tissue was performed. The primary antibody was Rabbit polyclonal antibody to Beta-2 Adrenergic Receptor (PA3116, Ambar). The fluorescent secondary antibodies were Cy3-conjugated goat anti-rabbit IgG (Powerful Biology, Wuhan, China). Additionally, 4’,6-diamidino-2-phenylindole (DAPI) (Powerful Biology, China) was used to stain the nuclei. Images were acquired by an Olympus FV1200 Laser Scanning Microscope and analyzed by CaseViewer 7.2.

### RNA-seq analysis

As previously described (6), results of RNA-sequence represented one experiment including 11 samples totally and n=3-4 in each groups. This project was based on the sequencing analysis platform of BGI Genomics Co Ltd. Bowtie2 was used to align clean reads to the genome sequence, and then used RSEM (a software package for RNA-seq reads to calculate gene and transcript subtype expression levels) to calculate the gene expression level of each sample. A supplementary file containing the RPKM values for all significantly changed genes was provide as an excel file (**Supplementary Material**).

### Statistical analysis

All data were analyzed using Prism 8.0 software (GraphPad Software, CA, USA). Data were expressed as mean ± standard error of the mean (SEM). Differences between two groups were analyzed using an unpaired two-tailed Student’s t test. Statistical analyses of multiple comparisons among three groups were performed using one-way ANOVA with Bonferroni’s or Tukey’s posthoc test, P<0.05 was considered to be statistically significant.

## Results

### Antibiotic treatment relieves allergic asthma

To investigate the effect of antibiotics on OVA-induced asthma and OT mouse models, antibiotics including neomycin sulfate, ampicillin sodium, vancomycin hydrochloride, and metronidazole were administered to post-weaning mice in drinking water for two weeks (**Figure 1A**). Both antibiotic administration and OVA treatment resulted in slightly slower weight gain in mice (**Figure 1B**). At the end point of models, cardiac blood and bronchoalveolar lavage fluid (BALF) were collected for measuring the immunoglobulin E (IgE) levels, which is an important marker of allergic asthma. We found that plasma and BALF IgE levels were significantly elevated in the asthmatic mice compared to that in the control and OT mice. However, the IgE levels were significantly reduced in asthmatic mice after antibiotic treatment (**Figures 1C, D**). Cell numbers and protein levels in BALF are also markers of inflammatory responses; we found that they were also significantly decreased in the asthmatic mice after antibiotic treatment (**Figures 1E, F**). In addition, antibiotic treatment reduced Th2 cells and relative gene expression levels of inflammatory factor including *Il4, Il5, Il13, Il10* and *Il33* in the asthmatic mice (**Figures 1G–M**). These results suggest that antibiotic treatment relieves allergic asthma.

### iNKT cells are decreased after antibiotic treatment

**Figure 2:**
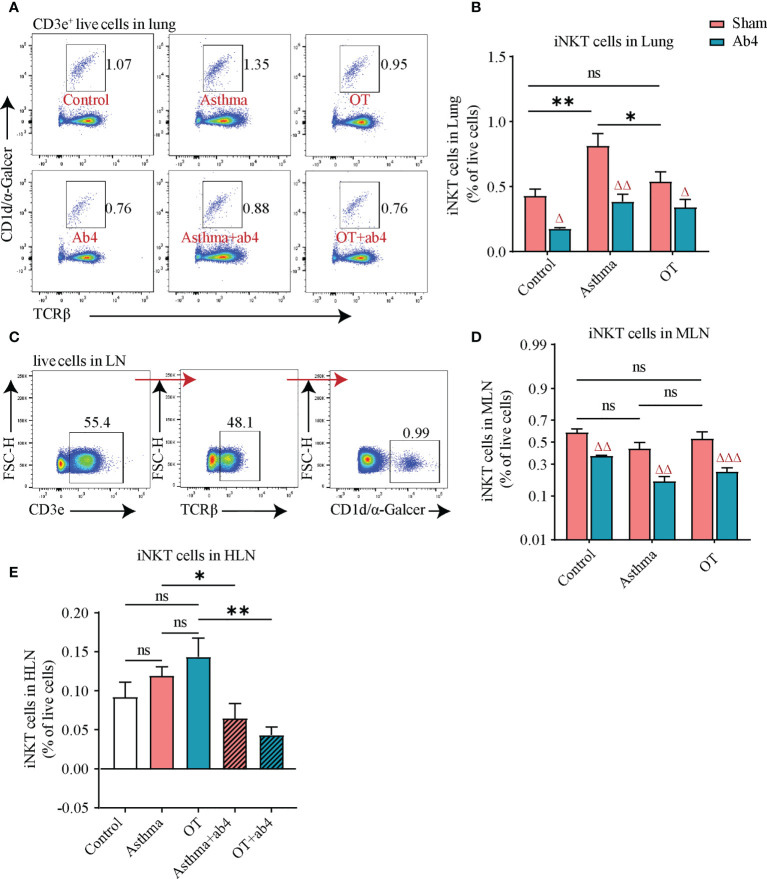
iNKT cell numbers are decreased after antibiotic treatment. **(A)** Flow cytometry gating strategy for CD3e^+^TCRβ^+^α-Galcer/CD1d tetramer^+^ iNKT cells in lung tissue. **(B)** Flow cytometry analysis for iNKT cells in lung tissue, **(C)** Flow cytometry gating strategy for iNKT cells in LN. Flow cytometry analysis for **(D)** iNKT cells in MLN, and **(E)** iNKT cells in HLN. All bars in this figure indicate mean ± SEM. * P < 0.05 and ** P < 0.01 represent multiple comparisons of the control, asthma and OT groups (corrected by one-way ANOVA with Tukey’s test). ^△^ P < 0.05, ^△△^ P < 0.01 and ^*△△△*^ P < 0.001 represent comparisons of adjacent bars of Ab4 and sham in each group (unpaired two-tailed Student’s t-test). **(A–D)**, data were pooled from 3 independent experiments, N = 3-4 in each independent experiments). * P < 0.05, **P < 0.01. (unpaired two-tailed Student’s t-test) **(E)**, data were pooled from 2 independent experiments, N = 3-4 in each independent experiments); ns, not significant.

Flow cytometry analysis of lung tissue showed that iNKT cells were significantly increased in asthmatic lungs compared to those in the control and OT lungs. However, they were significantly reduced in control, asthmatic and OT lungs after treatment with antibiotics (**Figures 2A, B**). Although there was no significant difference among the control, asthma and OT Lymph Nodes (LNs), iNKT cells were significantly reduced in the control, asthma and OT LNs post antibiotic treatment (**Figures 2C–E**). It is worth mentioning that the hilar lymph nodes (HLNs) of the control mice with antibiotic treatment were too small to obtain. These results suggest that antibiotic treatment may relieve allergic asthma accompanied with reduced frequencies of iNKTs in lung tissue and LNs.

### *Adrb2* and *Adrb3* genes are upregulated in asthmatic lungs after antibiotic treatment

**Figure 3:**
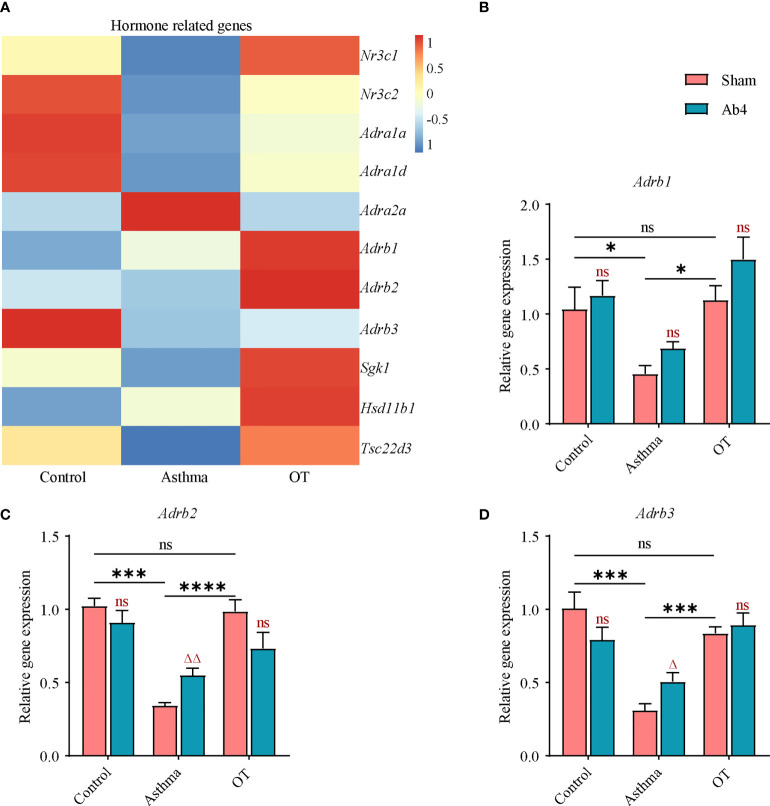
*Adrb2* and *Adrb3* genes are upregulated in asthmatic lungs after antibiotic treatment. **(A)** Heatmap showing the expression values (row normalization) of hormone-related genes in the control, asthmatic and OT lungs. The horizontal and vertical axes show the grouping information, and the gene name respectively (N = 3-4). **(B–D)** Gene expression levels of *Adrb1, Adrb2* and *Adrb3* in the whole lung of control, asthma and OT mice (with or without antibiotic treatment) were assessed by real-time qPCR (N = 4-5). Results are from one representative experiment of at least two independent experiments. All bars in this figure indicate mean ± SEM. * P < 0.05, *** P < 0.001, **** P < 0.0001 represent multiple comparisons of the control, asthma and OT groups (corrected by one-way ANOVA with Tukey’s test). ^△^ P < 0.05 and ^△△^ P < 0.01 represent comparisons of adjacent bars of Ab4 and sham in each group (unpaired two-tailed Student’s t-test); ns, not significant.

To further explore the effects of antibiotic treatment on asthma and OT mouse models, we first analyzed RNA-seq data from the control, asthmatic and OT lungs. Eleven hormone-related genes were identified to be differentially expressed among each group, as shown in the heatmap below (**Figure 3A**). Considering that β2-adrenergic receptors (ADRB2) not only affect bronchial smooth muscle relaxation but might also regulate inflammatory response (25), we looked at the expression levels of *Adrb* in lung tissue. We found that consistent with the results of RNA-seq, the expression levels of *Adrb1*, *Adrb2* and *Adrb3* were significantly decreased in asthmatic lungs compared with control and OT lungs. We also show that antibiotic treatment significantly further upregulated expression levels of *Adrb2* and *Adrb3* in asthmatic lungs (**Figures 3B–D**). These results hint that antibiotic treatment may relieve allergic asthma via regulating expression levels of ADRB in immune cells.

### Propranolol treatment alleviates allergic asthma

**Figure 4:**
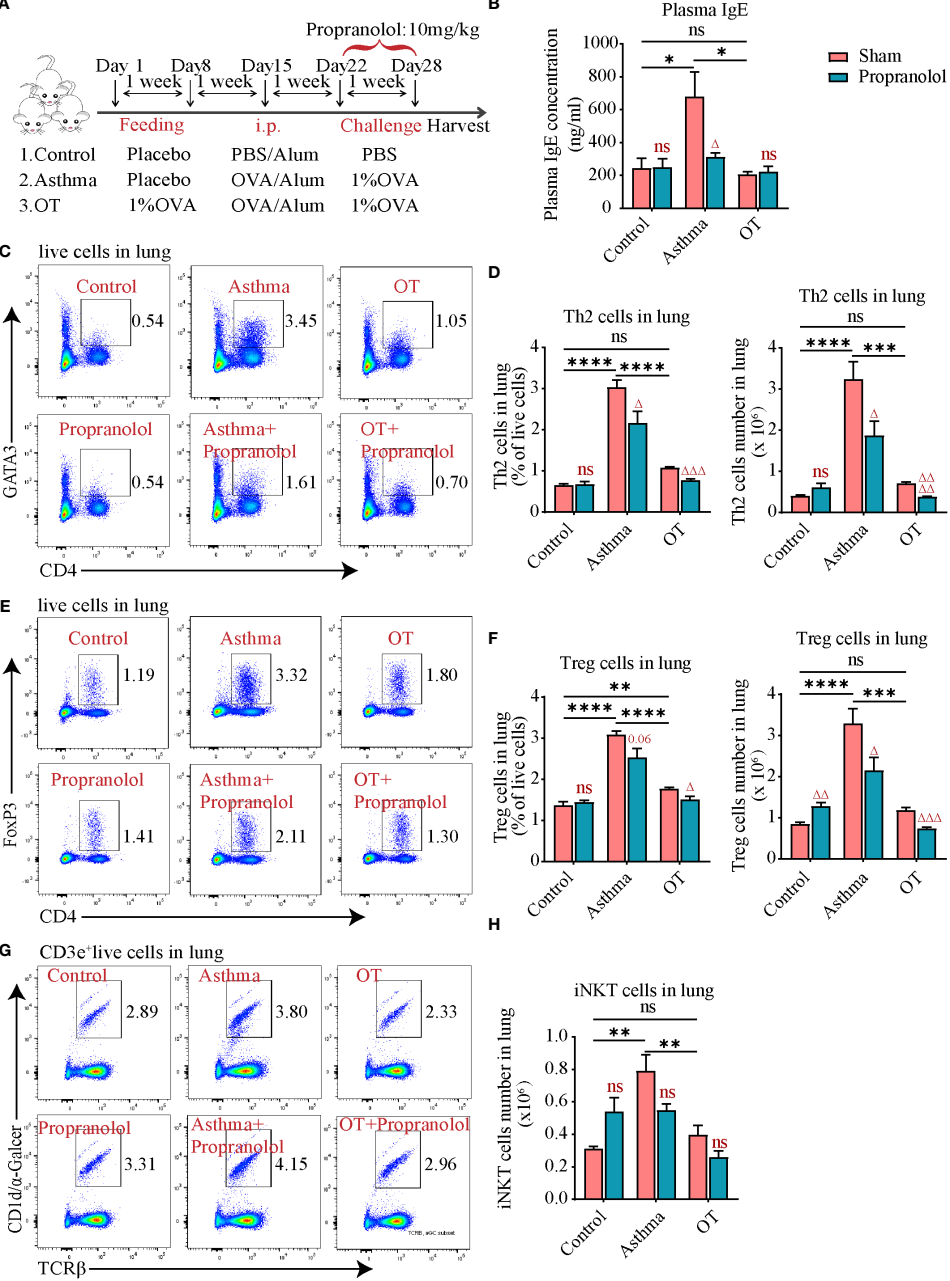
Propranolol treatment alleviates allergic asthma. **(A)** Experimental protocol for establishing asthma and OT mouse models with or without Propranolol treatment. The detailed procedures have been described in the “Materials and Methods”. **(B)** Plasma IgE levels assessed by ELISA. **(C, D)** Flow cytometry analysis for CD4^+^GATA3^+^ Th2 cells in lung tissue. **(E, F)** Flow cytometry analysis for CD4^+^FoxP3^+^ Treg cells in lung tissue. **(G, H)** Flow cytometry analysis for iNKT cells in lung tissue. Results are from one representative experiment of at least two independent experiments (N = 4-5). All bars in this figure indicate mean ± SEM. * P < 0.05, ** P < 0.01, *** P < 0.001, **** P < 0.0001 represent multiple comparisons of the control, asthma and OT groups (corrected by one-way ANOVA with Tukey’s test). ^△^ P < 0.05, ^△△^ P < 0.01, ^△△△^ P < 0.001 and ^△△△△^ P < 0.0001 represent comparisons of adjacent bars of Propranolol and sham in each group (unpaired two-tailed Student’s t-test); ns, not significant.

In order to clarify the role of ADRB2, propranolol, a non-selective blocker of ADRB1 and ADRB2 was chosen. As shown in **Figure 4A**, mice were given intraperitoneal injection of propranolol at 10 mg/kg 30 min before daily challenge (**Figure 4A**). Surprisingly, the administration of propranolol did not aggravate allergic asthma but decreased plasma IgE levels in asthmatic mice (**Figure 4B**). Further flow cytometry showed that Th2 cells (**Figures 4C, D**) and Treg cells (**Figures 4E, F**) were also significantly reduced in the asthmatic and OT lungs after propranolol treatment. However, the number of iNKT cells was not affected by the use of propranolol (**Figures 4G, H**). These results imply that activation of ADRB2 may be associated with the development of asthma in post-weaning mice.

### ADRB2 at protein level is upregulated in asthmatic lungs

**Figure 5:**
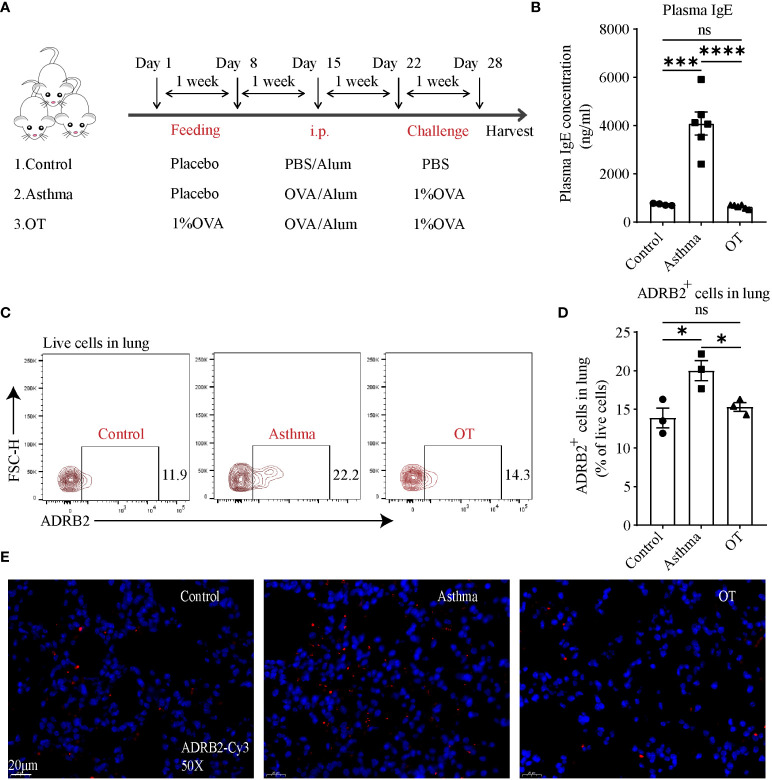
ADRB2 expression at protein level is upregulated in asthmatic lungs. **(A)** Experimental protocol for establishing asthma and OT mouse models. **(B)** Plasma IgE levels assessed by ELISA (N = 4-6). **(C, D)** Flow cytometry analysis for ADRB2^+^ cells in lung tissue (N = 3). **(E)** Representative confocal images of lung tissue with single-labeled ADRB2-Cy3 (red) and DAPI (blue) in objective magnification ×50 (N = 3). Scale bar represents 20μm. Results are from one representative experiment of at least two independent experiments. All bars in this figure indicate mean ± SEM. * P < 0.05, *** P < 0.001, **** P < 0.0001(unpaired two-tailed Student’s t-test); ns, not significant.

To further elucidate the changes in ADRB2 expression, we first established asthma and OT mouse models without antibiotic treatment (**Figure 5A**) and validated the model using plasma IgE levels (**Figure 5B**). We further detected ADRB2^+^ cells by flow cytometry in lung tissue and found that ADRB2^+^ cells were significantly increased in the asthmatic lungs compared to those in the control and OT lungs (**Figures 5C, D**). We also single-labeled ADRB2 by immunofluorescence in lung tissue and found that ADRB2 expression was significantly higher in asthmatic lungs compared to that in the control and OT lungs (**Figure 5E**). These results suggest that increased ADRB2 signaling may promote the development of asthma in post-weaning mice.

### ADRB2^+^ DCs in asthmatic lungs are downregulated after treatment with ICI118551

**Figure 6:**
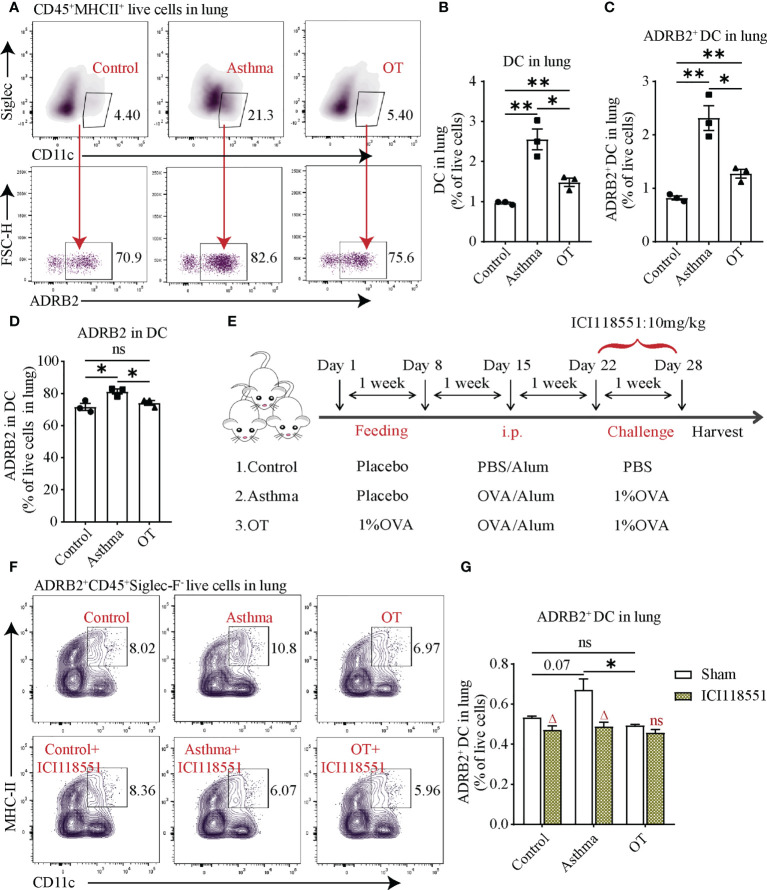
ADRB2^+^ DCs in asthmatic lungs are downregulated after treatment with ICI118551. **(A)** Flow cytometry gating strategy for ADRB2^+^CD45^+^Sigelc-F-CD11c^+^MHCII^+^ DCs in lung tissue. Flow cytometry analysis for **(B)** DCs, **(C)** ADRB2^+^ DCs, and **(D)** ADRB2 in DCs in lung tissue from the control, asthmatic, and OT lungs. **(E)** Experimental protocol for establishing asthma and OT mouse models with or without ICI118551 treatment. **(F, G)** Flow cytometry analysis for ADRB2^+^ DCs in lung tissue after treatment with ICI118551. Results are from one representative experiment of at least two independent experiments. All bars in this figure indicate mean ± SEM. * P < 0.05, **P < 0.01 (unpaired two-tailed Student’s t-test). **(A–D)**, N = 3); ns, not significant. * P < 0.05 represent multiple comparisons of control, asthma and OT groups (corrected by one-way ANOVA with Tukey’s test) and ^△^P < 0.05 represent comparisons of adjacent bars of ICI118551 and sham in each group (unpaired two-tailed Student’s t-test) **(E–G)**, N = 3-4); ns, not significant.

Given that both B and T cells were affected after propranolol treatment, we were interested to know whether DCs are affected due to the blockade of ADRB. DCs are capable of presenting antigens and are an important starting point for adaptive immunity (32, 33). We assessed DCs using flow cytometry and found that the number of DCs and ADRB2^+^ DCs was significantly higher in asthmatic lungs than those in the control and OT lungs (**Figures 6A–C**). Flow cytometry analysis also showed that more than 60% of DCs expressed ADRB2 in each group (**Figure 6D**). In addition, ADRB2^+^ DCs in the control and asthmatic lungs were significantly reduced after ICI118551 treatment (**Figures 6E–G**). These results indicate that DCs in the lungs of asthmatic mice express ADRB2, thus DCs may promote the development of asthma in post-weaning mice.

## Discussion

The use of antibiotics in asthma treatment is a debated research area. Here, we demonstrated that using antibiotics such as neomycin sulfate, ampicillin sodium, vancomycin hydrochloride, and metronidazole relieved OVA-induced allergic asthma in terms of IgE levels and inflammatory responses in post-weaning mice. We focused on iNKT cells, as they are similar to Th2 cells, and also because they secreted cytokines that can promote asthma inflammation in different ways (41). We also know that the development of iNKT cells depends on normal gut flora (18). Here, we clearly demonstrated that antibiotic treatment led to a reduction in iNKT cell numbers in lung tissue, HLNs and MLNs.

The activation of ADRB2 on human peripheral blood lymphocytes enhances IL-13 production (42), and studies have found that ADRB2 is required for mucus metaplasia, AHR and lung inflammatory cells in asthma models (43), and that the prolonged use of ADRB2 blockers could relieve allergic asthma (44, 45). However, the relationship between ADRB2 and asthma are not well-explained yet. Using RNA-seq analysis, we showed that the expression levels of some hormone-related genes, including *Adrb1, Adrb2* and *Adrb3* were downregulated in asthmatic lungs compared to that in the control and OT lungs. We further showed using qPCR that *Adrb2* and *Adrb3* were upregulated in asthmatic lungs after antibiotic treatment. Surprisingly, we also found that propranolol relieved allergic asthma symptoms. To further confirm the expression levels and function of ADRB2, flow cytometry and immunofluorescence were used. We found that asthmatic lungs had much higher levels of ADRB2 compared to the control and OT lungs. These results suggest that ADRB2 may play a role in promoting the development of asthma. Therefore, it is important to identify the cell types that express ADRB2 and how they function in alleviating allergic asthma.

ADRB2 is associated with a variety of immune cells. Memory CD8^+^ T cells express ADRB2, which in turn produce more inflammatory cytokines post norepinephrine (NE) treatment (46). In addition, ADRB2^+^ DCs are also known to be associated with inflammation (35–37). Marrow-derived DCs exposed to NE induce a Th2 response and limit the polarizing properties of Th1 (47). Other studies have also reported that the production of IL-33 was enhanced in DCs with NE treatment, which was completely blocked by ADRB2 specific antagonist ICI118,551 (48). In this study, we demonstrated that DCs, especially ADRB2^+^ DCs were increased in asthmatic lungs and reduced after ICI18551 treatment, indicating that DCs expressing ADRB2 might be associated with inflammatory response in asthma.

A disadvantage of this study is that we have not yet established a link between antibiotic treatment and changes in ADRB2^+^ DCs. We have to note that several studies have shown that antibiotics can modulate dendritic cell function. For example, azithromycin inhibits dendritic cell-induced allogeneic T cell proliferation and cytokine production (49). In terms of dendritic cell expression of the signaling molecule TRAF6 as a non-redundant requirement for maintaining immune tolerance in the mouse small intestine, antibiotic-treated specific pathogen-free mice in the absence of dendritic cell-expressed TRAF6 exhibited restored Immune tolerance (50). Amoxicillin induces increased phosphorylation of three MAPKs and activation of NF-κB in DCs from allergic patients. Furthermore, inhibition of these pathways prevents amoxicillin-induced upregulation of surface molecules (51). More importantly, vancomycin is able to alter the microbiome and metabolite profile, such as short-chain fatty acids, which can modulate T cell and dendritic cell activity (52). All these findings in references may help us to explain why antibiotics affect ADRB2^+^ DCs.

In this study, we found that antibiotic therapy can increase the expression of *Adrb2*. ADRB2 is expressed in bronchial smooth muscle cells (53, 54) and bronchial epithelial cells, which contribute more RNA than dendritic cells during RNAseq. Testosterone has been reported to enhance β2 adrenergic receptor genome transcription, thereby increasing salbutamol relaxation in airway smooth muscle (55). Given that feedback regulation plays a crucial role in dynamic gene expression in nature (56), we hypothesized that antibiotic treatment might increase *Adrb2* expression at the mRNA level in smooth muscle cells. Antibiotics may reduce ADRB2 protein expression in dendritic cells, but increase their mRNA levels. In fact, ADRB2 is a causative factor in asthma because ADRB2^+^ DCs were significantly increased in asthma but decreased in OT mice. Blocking ADRB2 with its blocker propranolol can alleviate asthma, and its antagonist ICI118551 can reduce ADRB2^+^ DCs in asthmatic mice. Therefore, we conclude that antibiotic treatment reduces asthma possibly due to dysfunction of ADRB2.In conclusion, we showed that antibiotic treatment for 2 weeks in post-weaning mice relieved OVA-induced allergic asthma, accompanied with reduced frequency of iNKT cells and increased expressions of *Adrb2*. Additionally, ADRB2^+^ DCs were increased in asthmatic lungs and might be involved in promoting the development of allergic asthma. Our research reveals the mechanisms underlying the development of asthma and provides new ideas for the treatment of asthma and the establishment of OT. We will further explore what has changed in the gut microbiota after using oral antibiotics and identify microbiota or metabolites that play a role in relieving allergic asthma.

## Data availability statement

An excel file containing the RPKM values for all significantly changed genes was provided as Supplementary Material. The other raw data supporting the conclusions of this article will be made available by the authors on request.

## Ethics statement

The animal study was reviewed and approved by the Laboratory Animal Welfare Ethics Committee of the Pasteur Institute of Shanghai, Chinese Academy of Sciences.

## Author contributions

XS and ZJ conceived and designed the experiments and edited the manuscript. NL performed the experiments, analyzed data, and wrote the original draft. JC and SX reviewed the manuscript and assisted with establishment of models and data duration. MZ, TS, and YH assisted with animal experiments and data duration. All authors contributed to the manuscript and agreed to the final submitted version.

## Funding

This work was supported by National Natural Science Foundation of China (No.82070024, No.91942305, and No.81970075) and Natural Science Foundation Project of “Science and Technology Innovation Action Plan” of Shanghai in 2021 (20ZR1443700), Science and Technology Commission of Shanghai Municipality (20DZ2261200), and Innovative research team of high-level local universities in Shanghai (SHSMU-ZDCX20210602). The principal funding recipients are XS and ZJ.

## Conflict of interest

The authors declare that the research was conducted in the absence of any commercial or financial relationships that could be construed as a potential conflict of interest.

The handling editor XZ declared a past collaboration with the author XS.

## Publisher’s note

All claims expressed in this article are solely those of the authors and do not necessarily represent those of their affiliated organizations, or those of the publisher, the editors and the reviewers. Any product that may be evaluated in this article, or claim that may be made by its manufacturer, is not guaranteed or endorsed by the publisher.
